# Supplementary material for: Subthalamic beta-targeted neurofeedback speeds up movement initiation but increases tremor in Parkinsonian patients
Source: eLife. 2020 Nov 18;9:e60979. doi: 10.7554/eLife.60979 (PMC7695453; doi:10.7554/eLife.60979)
Supplement: Source code 1. [file elife-60979-code1.zip › eLife_code_data_eLife-60979_04Nov2020/kakearney-boundedline-pkg-8179f9a/README.html]

boundedline.m: line with shaded error/confidence bounds 

# boundedline.m: line with shaded error/confidence bounds

Author: Kelly Kearney

This repository includes the code for the boundedline.m Matlab function and the accompanying outlinebounds.m function, along with all dependent functions required to run them.

The boundedline function allows a user to easily plot and line with a shaded patch around it. Ths sort of plot is often used to indicate uncertainty intervals or error bounds around a line.

## Contents

- Getting started
- Syntax
- Example 1: Plotting lines using various syntax options
- Example 2: Filling gaps
- Contributions

## Getting started

**Prerequisites**

This function requires Matlab R14 or later.

**Downloading and installation**

This code can be downloaded from Github or the MatlabCentral File Exchange. The File Exchange entry is updated daily from the GitHub repository.

**Matlab Search Path**

The following folders need to be added to your Matlab Search path (via addpath, pathtool, etc.):

```
boundedline-pkg/Inpaint_nans
boundedline-pkg/boundedline
boundedline-pkg/catuneven
boundedline-pkg/singlepatch
```

## Syntax

boundedline(x, y, b) plots a line with coordinates given by x and y, surrounded by a patch extending a certain distance b above/below that line. The dimensions of the x, y, and b arrays can vary to allow for multiple lines to be plotted at once, and for patch bounds to be either constant or varying along the length of the line. See function header help for full details of these variations.

boundedline(..., 'alpha') renders the bounded area patch using a partially-transparent patch the same color as the corresponding line(s). If not included, the bounded area will use a fully-opaque patch in a lighter shade of the corresponding line color.

boundedline(..., 'transparency', transp) indicates the tranparency or intensity of the bounds patch, using a scalar between 0 and 1. Default is 0.2.

boundedline(..., 'orientation', orient) indicates the orientation of the bounds. Orientation can be either 'vert' for vertical (y-direction) bounds, or 'horiz' for horizontal (x-direction) bounds. Default is 'vert'.

boundedline(..., 'nan', nanflag) indicates how the bounds patch should handle NaNs in the line coordinates or bounds values. Options are 'fill', to smooth over the gap using neighboring values, 'gap' to leave a blank space in the patch at those points, or 'remove' to drop the NaN-points entirely, leading to linear interpolation of the gap in the patch. See examples below for more details on these options.

boundedline(..., 'cmap', cmap) colors the lines (in order of plotting) acording to the colors in this n x 3 colormap array, overriding any linespec or default colors.

boundedline(..., ax) plots the bounded line to the axis indicated by handle ax. If not included, the current axis is used.

[hl, hp] = boundedline(...) returns the handles the resulting line and patch object(s).

hout = outlinebounds(hl, hp) adds an outline to the bounds patch generated by boundedline, returning the handle of the resulting line object in hout.

Full details of all input and output variables for both functions can be accessed via the help function.

## Example 1: Plotting lines using various syntax options

This example builds the 4-panel example image used on the MatlabCentral File Exchange, which shows several different methods for supplying line coordinates, bounds coordinates, and shading options.

The first axis plots two lines using the LineSpec option for input, which allows yoy to set line color, line color, and marker type for each line. The bounds on the first line vary over x, while the bounds on the second line are constant for all x. An outline is added to the bounds so the overlapping region can be seen more clearly.

```
x = linspace(0, 2*pi, 50);
y1 = sin(x);
y2 = cos(x);
e1 = rand(size(y1))*.5+.5;
e2 = [.25 .5];

ax(1) = subplot(2,2,1);
[l,p] = boundedline(x, y1, e1, '-b*', x, y2, e2, '--ro');
outlinebounds(l,p);
title('Opaque bounds, with outline');
axis tight;
```

For our second axis, we use the same 2 lines, and this time assign x-varying bounds to both lines. Rather than using the LineSpec syntax, this example uses the default color order to assign the colors of the lines and patches. I also turn on the 'alpha' option, which renders the patch wit partial transparency.

```
ax(2) = subplot(2,2,2);
boundedline(x, [y1;y2], rand(length(y1),2,2)*.5+.5, 'alpha');
title('Transparent bounds');
axis tight;
```

The bounds can also be assigned to a horizontal orientation, for a case where the x-axis represents the dependent variable. In this case, the scalar error bound value applies to both lines and both sides of the lines.

```
ax(3) = subplot(2,2,3);
boundedline([y1;y2], x, e1(1), 'orientation', 'horiz')
title('Horizontal bounds');
axis tight;
```

Rather than use a LineSpec or the default color order, a colormap array can be used to assign colors. In this case, increasingly-narrower bounds are added on top of the same line.

```
ax(4) = subplot(2,2,4);
boundedline(x, repmat(y1, 4,1), permute(0.5:-0.1:0.2, [3 1 2]), ...
'cmap', cool(4), ...
'transparency', 0.5);
title('Multiple bounds using colormap');

set(ax([1 2 4]), 'xlim', [0 2*pi]);
set(ax(3), 'ylim', [0 2*pi]);
axis tight;
```

## Example 2: Filling gaps

If you plot a line with one or more NaNs in either the x or y vector, the NaN location is rendered as a missing marker with a gap in the line. However, the patch command does not handle NaNs gracefully; it simply fails to show the patch at all if any of the coordinates include NaNs.

Because of this, the expected behavior of the patch part of boundedline when confronted with a NaN in either the bounds array (b) or the x/y-coordinates of the line (which are used to calculate the patch coordinates) is ambiguous. I offer a few options.

Before I demonstrate the options, I'll create a dataset that has a few different types of gaps:

```
x = linspace(0, 2*pi, 50);
y = sin(x);
b = [ones(size(y))*0.2; rand(size(y))*.5+.5]';

y(10)   = NaN;  % NaN in the line but not bounds
b(20,1) = NaN;  % NaN in lower bound but not line
b(30,2) = NaN;  % NaN in upper bound but not line
b(40,:) = NaN;  % NaN in both sides of bound but not line
```

Here's what that looks like in an errorbar plot.

```
figure;
he = errorbar(x,y,b(:,1), b(:,2), '-bo');


line([x([10 20 30 40]); x([10 20 30 40])], [ones(1,4)*-2;ones(1,4)*2], ...
'color', ones(1,3)*0.5, 'linestyle', ':');
text(x(10), sin(x(10))-0.2, {'\uparrow','Line','gap'}, 'vert', 'top', 'horiz', 'center');
text(x(20), sin(x(20))-0.2, {'\uparrow','Lower','bound','gap'}, 'vert', 'top', 'horiz', 'center');
text(x(30), sin(x(30))-0.2, {'\uparrow','Upper','bound','gap'}, 'vert', 'top', 'horiz', 'center');
text(x(40), sin(x(40))-0.2, {'\uparrow','Two-sided','bound','gap'}, 'vert', 'top', 'horiz', 'center');

axis tight equal;
```

The default method for dealing with NaNs in boundedline is to leave the gap in the line, but smooth over the gap in the bounds based on the neighboring points. This option can be nice if you only have one or two missing points, and you're not interested in emphasizing those gaps in your plot:

```
delete(he);
[hl,hp] = boundedline(x,y,b,'-bo', 'nan', 'fill');
ho = outlinebounds(hl,hp);
set(ho, 'linestyle', ':', 'color', 'r', 'marker', '.');
```

I've added bounds outlines in a contrasting color so you can see how I'm handling individual points.

The second option leaves a full gap in the patch for any NaN. I considered allowing one-sided gaps, but couldn't think of a good way to distinguish a gap from a zero-valued bound. I'm open to suggestions if you have any (email me).

```
delete([hl hp ho]);
[hl,hp] = boundedline(x,y,b,'-bo', 'nan', 'gap');
ho = outlinebounds(hl,hp);
set(ho, 'linestyle', ':', 'color', 'r', 'marker', '.');
```

The final option removes points from the patch that are NaNs. The visual result is very similar to the fill option, but the missing points are apparent if you plot the bounds outlines.

```
delete([hl hp ho]);
[hl,hp] = boundedline(x,y,b,'-bo', 'nan', 'remove');
ho = outlinebounds(hl,hp);
set(ho, 'linestyle', ':', 'color', 'r', 'marker', '.');
```

## Contributions

Community contributions to this package are welcome!

To report bugs, please submit an issue on GitHub and include:

- your operating system
- your version of Matlab and all relevant toolboxes (type ver at the Matlab command line to get this info)
- code/data to reproduce the error or buggy behavior, and the full text of any error messages received

Please also feel free to submit enhancement requests, or to send pull requests (via GitHub) for bug fixes or new features.

I do monitor the MatlabCentral FileExchange entry for any issues raised in the comments, but would prefer to track issues on GitHub.

Published with MATLAB® R2016b
