# Supplementary figures and images for: Subthalamic beta-targeted neurofeedback speeds up movement initiation but increases tremor in Parkinsonian patients
Source: eLife. 2020 Nov 18;9:e60979. doi: 10.7554/eLife.60979 (PMC7695453; doi:10.7554/eLife.60979)

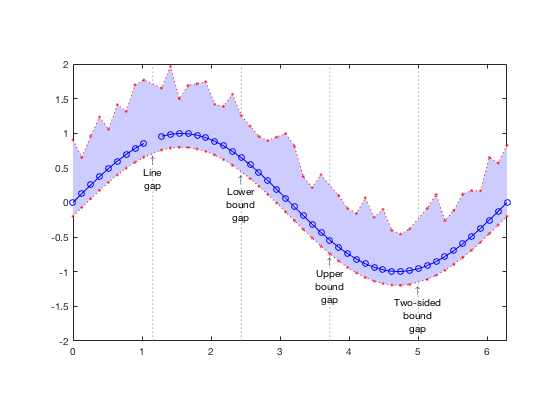

Supplement: Source code 1. [file elife-60979-code1.zip › eLife_code_data_eLife-60979_04Nov2020/kakearney-boundedline-pkg-8179f9a/readmeExtras/README_08.png]

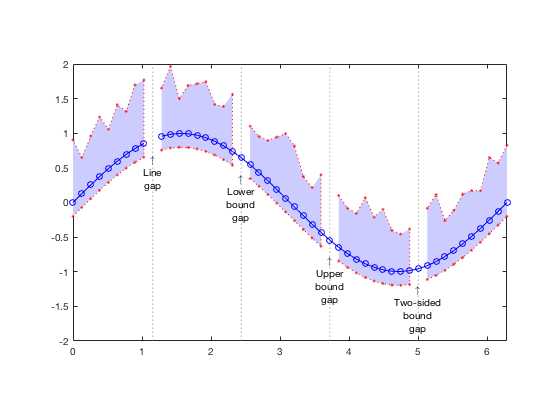

Supplement: Source code 1. [file elife-60979-code1.zip › eLife_code_data_eLife-60979_04Nov2020/kakearney-boundedline-pkg-8179f9a/readmeExtras/README_07.png]

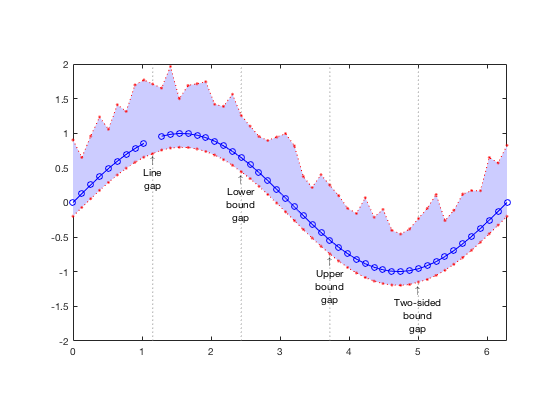

Supplement: Source code 1. [file elife-60979-code1.zip › eLife_code_data_eLife-60979_04Nov2020/kakearney-boundedline-pkg-8179f9a/readmeExtras/README_06.png]

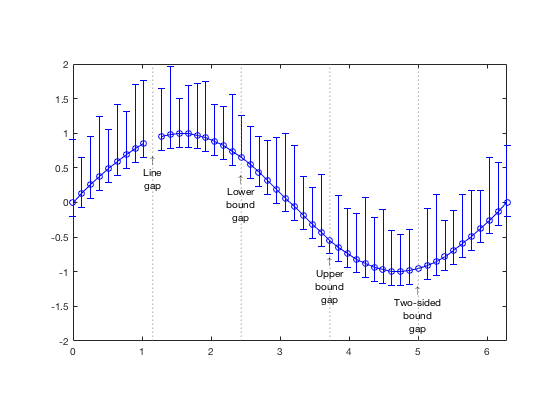

Supplement: Source code 1. [file elife-60979-code1.zip › eLife_code_data_eLife-60979_04Nov2020/kakearney-boundedline-pkg-8179f9a/readmeExtras/README_05.png]

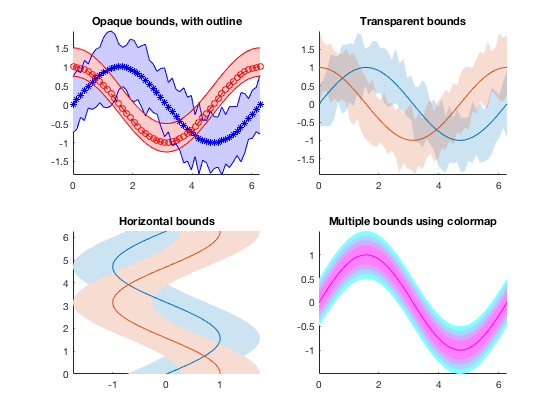

Supplement: Source code 1. [file elife-60979-code1.zip › eLife_code_data_eLife-60979_04Nov2020/kakearney-boundedline-pkg-8179f9a/readmeExtras/README_04.png]

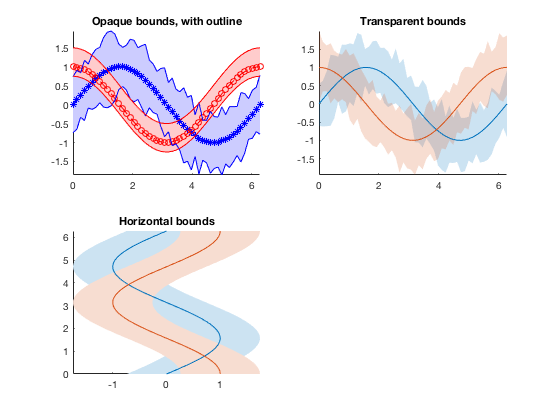

Supplement: Source code 1. [file elife-60979-code1.zip › eLife_code_data_eLife-60979_04Nov2020/kakearney-boundedline-pkg-8179f9a/readmeExtras/README_03.png]

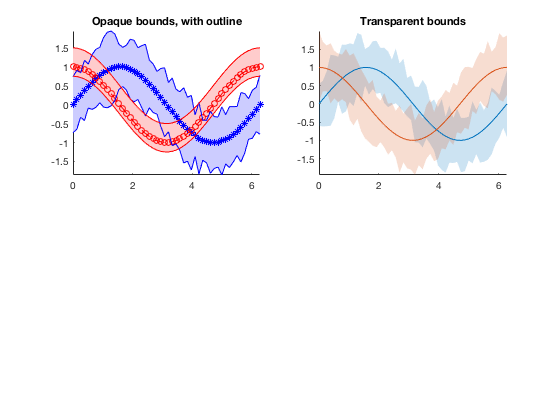

Supplement: Source code 1. [file elife-60979-code1.zip › eLife_code_data_eLife-60979_04Nov2020/kakearney-boundedline-pkg-8179f9a/readmeExtras/README_02.png]

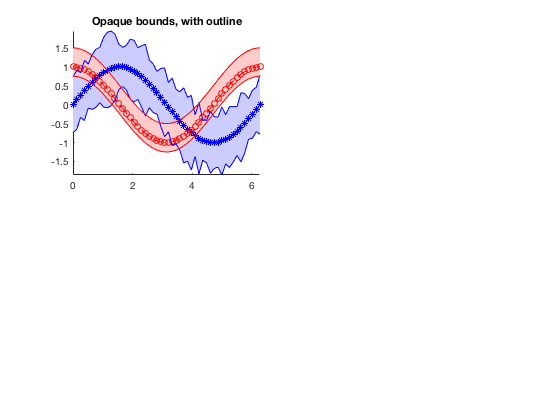

Supplement: Source code 1. [file elife-60979-code1.zip › eLife_code_data_eLife-60979_04Nov2020/kakearney-boundedline-pkg-8179f9a/readmeExtras/README_01.png]

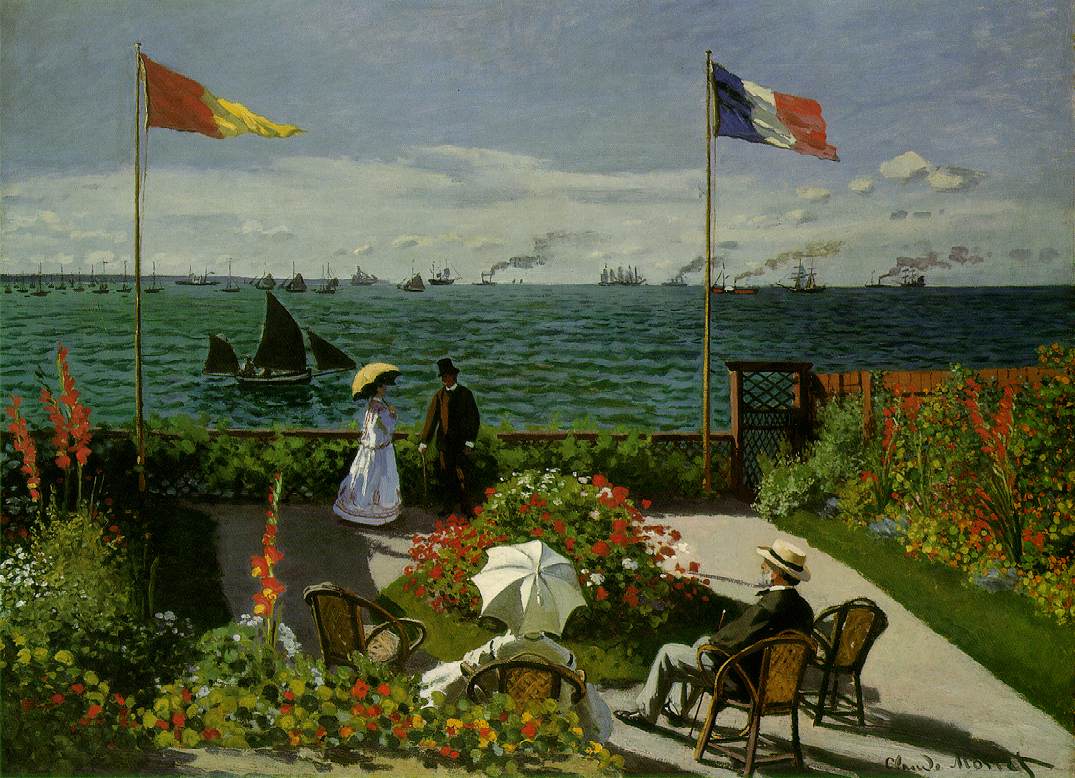

Supplement: Source code 1. [file elife-60979-code1.zip › eLife_code_data_eLife-60979_04Nov2020/kakearney-boundedline-pkg-8179f9a/Inpaint_nans/monet_adresse.jpg]

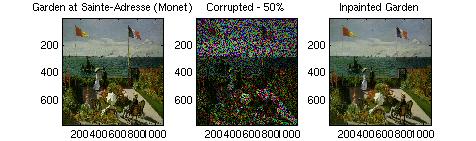

Supplement: Source code 1. [file elife-60979-code1.zip › eLife_code_data_eLife-60979_04Nov2020/kakearney-boundedline-pkg-8179f9a/Inpaint_nans/garden50.jpg]

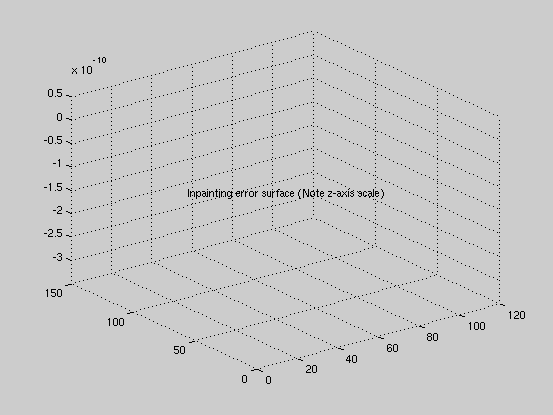

Supplement: Source code 1. [file elife-60979-code1.zip › eLife_code_data_eLife-60979_04Nov2020/kakearney-boundedline-pkg-8179f9a/Inpaint_nans/demo/html/inpaint_nans_demo_06.png]

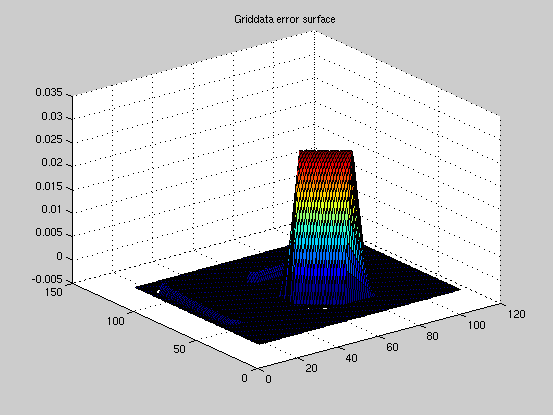

Supplement: Source code 1. [file elife-60979-code1.zip › eLife_code_data_eLife-60979_04Nov2020/kakearney-boundedline-pkg-8179f9a/Inpaint_nans/demo/html/inpaint_nans_demo_05.png]

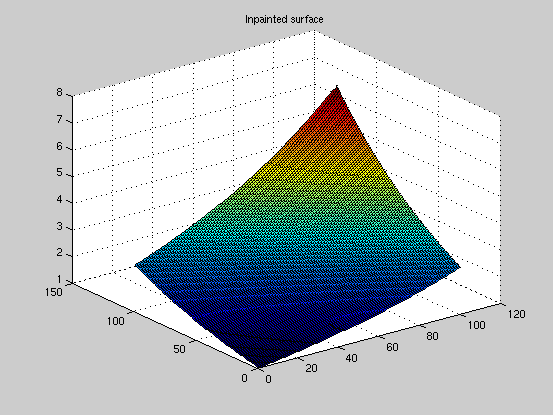

Supplement: Source code 1. [file elife-60979-code1.zip › eLife_code_data_eLife-60979_04Nov2020/kakearney-boundedline-pkg-8179f9a/Inpaint_nans/demo/html/inpaint_nans_demo_04.png]

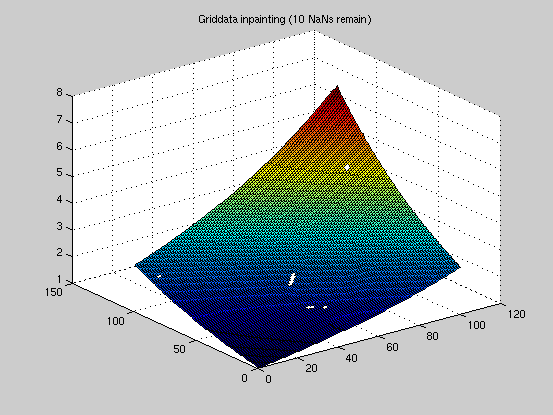

Supplement: Source code 1. [file elife-60979-code1.zip › eLife_code_data_eLife-60979_04Nov2020/kakearney-boundedline-pkg-8179f9a/Inpaint_nans/demo/html/inpaint_nans_demo_03.png]

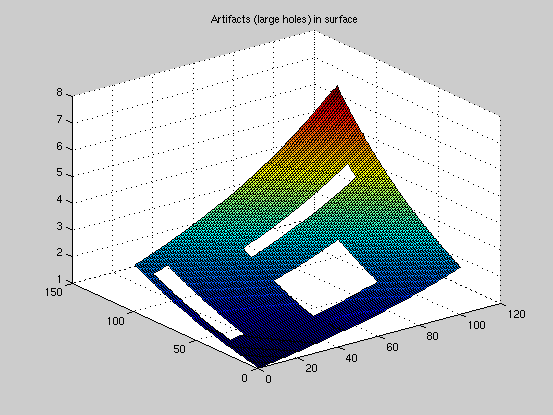

Supplement: Source code 1. [file elife-60979-code1.zip › eLife_code_data_eLife-60979_04Nov2020/kakearney-boundedline-pkg-8179f9a/Inpaint_nans/demo/html/inpaint_nans_demo_02.png]

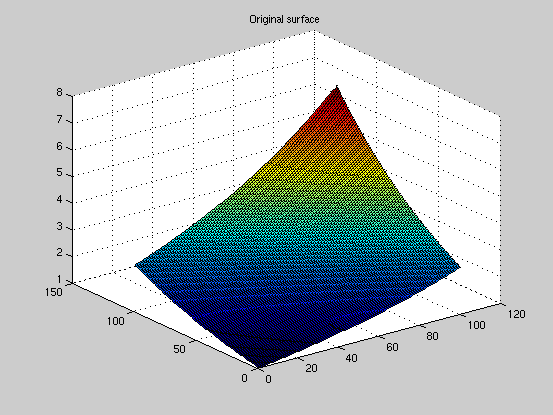

Supplement: Source code 1. [file elife-60979-code1.zip › eLife_code_data_eLife-60979_04Nov2020/kakearney-boundedline-pkg-8179f9a/Inpaint_nans/demo/html/inpaint_nans_demo_01.png]

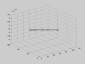

Supplement: Source code 1. [file elife-60979-code1.zip › eLife_code_data_eLife-60979_04Nov2020/kakearney-boundedline-pkg-8179f9a/Inpaint_nans/demo/html/inpaint_nans_demo.png]
