# Supplementary material for: Subthalamic beta-targeted neurofeedback speeds up movement initiation but increases tremor in Parkinsonian patients
Source: eLife. 2020 Nov 18;9:e60979. doi: 10.7554/eLife.60979 (PMC7695453; doi:10.7554/eLife.60979)
Supplement: Transparent reporting form [file elife-60979-transrepform.docx]

***eLife’s* transparent reporting form**

We encourage authors to provide detailed information *within their submission* to facilitate the interpretation and replication of experiments. Authors can upload supporting documentation to indicate the use of appropriate reporting guidelines for health-related research (see [EQUATOR Network](http://www.equator-network.org/%20)), life science research (see the [BioSharing Information Resource](https://biosharing.org/)), or the [ARRIVE guidelines](http://www.plosbiology.org/article/info:doi/10.1371/journal.pbio.1000412) for reporting work involving animal research. Where applicable, authors should refer to any relevant reporting standards documents in this form.

If you have any questions, please consult our Journal Policies and/or contact us: [editorial@elifesciences.org](mailto:editorial@elifesciences.org).

**Sample-size estimation**

- You should state whether an appropriate sample size was computed when the study was being designed
- You should state the statistical method of sample size computation and any required assumptions
- If no explicit power analysis was used, you should describe how you decided what sample (replicate) size (number) to use

Please outline where this information can be found within the submission (e.g., sections or figure legends), or explain why this information doesn’t apply to your submission:

In this study, we tried to investigate whether the patients with Parkinson’s disease can self-suppress beta oscillations in STN through neurofeedback training, and whether the suppression of STN beta has any impact on the forthcoming motor task. We also used the generalised multi-level modelling to investigate relationship between oscillatory activities in different frequency bands in the STN before a ‘Go-Cue’ and the reaction time.

Only two existing studies have been focused on STN beta-targeted neurofeedback training and both had relatively small sample-size. In Fukuma et al. 2018, 4 patients were trained to down-regulate STN beta, and all of them showed significant reduction of STN beta during the training. Fischer et al. (2017) found in average 10% of beta power reduction which was statistically significant (p<0.01) during imaginary movements with 10 participants. Considering the fact that post-operative recordings from deep brain stimulation electrodes are still rare research opportunities, we recruited 12 patients (21 STNs in total, **Table I**)

Another question is how many trials we would need to investigate trial-to-trial relationship between oscillatory STN activities and motor performance in each patient. MLM has been used in several previous studies. With the cluster number of 10, and the 40 observations per condition per cluster, and a middle effect size, the statistical power of the MLM is above 0.8 (Fig. 5 in Aarts et al, 2015; Danhier et al., 2013). Studies with Monte Carlo simulation showed that in continuous response multilevel models, the regression parameter estimates appear unbiased even with small sample size. Maas and Hox (2005) showed that only a small sample size at level two (meaning a sample of 50 or less) leads to biased estimates of the second-level standard errors; in all of the other simulated conditions the estimates of the regression coefficients, the variance components, and the standard errors are unbiased. Paccagnella (2011) showed that the accuracy of the standard errors of the regression parameter estimates is achieved with a number of groups equal to 50. In Tan et al. 2015, MLM helped to show that STN LFPs help to encode effort rather than absolute pressing force on data recorded from 11 patients with roughly 50 trials for pressing with index finger and 50 trials of pressing with little finger. In Toreccillos et al. 2018, MLM helped to show that beta bursts within a short window before the go-cue lead to slowing down in the movement speed based on recordings from roughly 12 patients with 50 trials per patient. In the current study, around 88 trials in total were recorded for each hemisphere (**L605**). Therefore, we assume this would be reasonably sufficient.

*Aarts E, Dolan CV, Verhage M, van der Sluis S (2015) Multilevel analysis quantifies variation in the experimental effect while optimizing power and preventing false positives. BMC neuroscience 16:94.*

*Danhier J, Giladi M, Veny Y (2013) Bias in multilevel modeling with small level-two sample size: A simulation study. Conference: 5th Conference of the European Survey Research Association (ESRA 2013) Ljubljana*

*Fischer P, Pogosyan A, Cheeran B, Green AL, Aziz TZ, Hyam J, Little S, Foltynie T, Limousin P, Zrinzo L, Hariz M (2017) Subthalamic nucleus beta and gamma activity is modulated depending on the level of imagined grip force. Exp Neurol 293:53-61.*

*Fukuma R, Yanagisawa T, Tanaka M, Yoshida F, Hosomi K, Oshino S, Tani N, Kishima H (2018) Real-time neurofeedback to modulate β-band power in the subthalamic nucleus in Parkinson’s disease patients. eNeuro 5.*

*Paccagnella O (2011) Sample size and accuracy of estimates in multilevel models. Methodology 7:111-120.*

*Maas CJ, Hox JJ (2005) Sufficient sample sizes for multilevel modeling. Methodology 1:86-92.*

*Tan H, Pogosyan A, Ashkan K, Cheeran B, FitzGerald JJ, Green AL, Aziz T, Foltynie T, Limousin P, Zrinzo L, Brown P (2015) Subthalamic nucleus local field potential activity helps encode motor effort rather than force in parkinsonism. J Neurosci 35:5941-5949.*

*Torrecillos F, Tinkhauser G, Fischer P, Green AL, Aziz TZ, Foltynie T, Limousin P, Zrinzo L, Ashkan K, Brown P, Tan H (2018) Modulation of beta bursts in the subthalamic nucleus predicts motor performance. J Neurosci 38:8905-8917.*

**Replicates**

- You should report how often each experiment was performed
- You should include a definition of biological versus technical replication
- The data obtained should be provided and sufficient information should be provided to indicate the number of independent biological and/or technical replicates
- If you encountered any outliers, you should describe how these were handled
- Criteria for exclusion/inclusion of data should be clearly stated
- High-throughput sequence data should be uploaded before submission, with a private link for reviewers provided (these are available from both GEO and ArrayExpress)

Please outline where this information can be found within the submission (e.g., sections or figure legends), or explain why this information doesn’t apply to your submission:

Our biological replicates, i.e. “parallel measurements of biologically distinct samples that capture random biological variation” as defined by Blainey et al (2014, Nature Methods), are the 21 recorded STN hemispheres from 12 patients.

The data is described in more detail in the Materials and Methods section, Table I, and Figure I. For example:

**L453-454:** Twelve Parkinsonian patients (4 females), who underwent bilateral implantation of DBS electrodes targeting the motor area of the STN, participated in this study.

**L502-503:** Each experimental session consisted of 30 seconds of rest, a block of 10 trials in the ‘Training’ condition and a block of 10 trials in the ‘No Training’ condition (Fig. 1B).

**L510-513:** Nine out of 12 participants completed 4 sessions of the task separately with both hemispheres and contralateral arms, and the other three participants only completed the task with the dominant hand for the motor task and the contralateral STN. All trials were visually inspected and those with obvious movement during the neurofeedback phase were excluded.

**L515-516:** Four patients repeated the same task over two consecutive days with both hemispheres, which allowed us to investigate overnight learning effects.

**L602-604:** Force measurements from individual trials were visually inspected; those trials with obvious artefacts, failed to pinch within 2 seconds after the Go-cue, or with a reaction time smaller than 0.2 s were excluded.

**L604-607:** Thus, for each of the 21 STN hemispheres we analysed 44.38 ± 3.88 (mean ± SEM) and 44.57 ± 3.84 trials in the ‘Training’ and ‘No Training’ conditions, respectively, resulting in 1868 trials in total across all tested hemispheres.

**Statistical reporting**

- Statistical analysis methods should be described and justified
- Raw data should be presented in figures whenever informative to do so (typically when N per group is less than 10)
- For each experiment, you should identify the statistical tests used, exact values of N, definitions of center, methods of multiple test correction, and dispersion and precision measures (e.g., mean, median, SD, SEM, confidence intervals; and, for the major substantive results, a measure of effect size (e.g., Pearson's r, Cohen's d)
- Report exact p-values wherever possible alongside the summary statistics and 95% confidence intervals. These should be reported for all key questions and not only when the p-value is less than 0.05.

Please outline where this information can be found within the submission (e.g., sections or figure legends), or explain why this information doesn’t apply to your submission:

**L662-667:** Paired t tests (Matlab function *ttest*) or nonparametric Wilcoxon signed-rank test (Matlab function *signrank*), depending on whether the normal distribution assumption was satisfied, were used to evaluate the effect of the experimental condition (‘Training’ and ‘No Training’) on neurofeedback task performance, the motor task reaction time, tremor severity, and neural activities measured in STN LFPs and EEGs. The normal distribution assumption was tested using Anderson-Darling test (Matlab function *adtest*) (Anderson and Darling, 1952).

**L667-668:** Multiple comparisons applied to different measurements were corrected using Bonferroni correction.

For each key comparison, t-values (z-values) and p-values were reported in the main text as well as in the figures.

**L647-649:** Generalised linear mixed effects modelling (GLME, Matlab function *fitglme*) was used to assess the trial-to-trial within subject relationship between different measurements, and how they were changed by neurofeedback training.

**L649-654:** Apart from transforming the dependent variable to eliminate the deviation from normality distribution, GLME also allows researchers to select a theoretical distribution that matches the properties of the dependent variable (Lo and Andrews, 2015). For example, the measured RT is skewed and closer to an Inverse Gaussian distribution instead of a normal Gaussian distribution, thus an Inverse Gaussian distribution was selected in the models using RT as dependent variable.

**L654-658:** When applying GLME modelling, data from all valid individual trials from all tested hemispheres were considered, and the average power (10log10 transferred to dB) were used when applicable. The slope(s) between the predictor(s) and the dependent variable were set to be fixed across all hemispheres; a random intercept was set to vary by hemisphere.

**L670-672:** When GLME modelling was used, the estimated fixed effect coefficient (*k*), which indicates the potential positive or negative correlation between the predictor and the dependent variable, the corresponding t-statistic *p*-value, and R^2^ were reported.

(For large datasets, or papers with a very large number of statistical tests, you may upload a single table file with tests, Ns, etc., with reference to sections in the manuscript.)

**Group allocation**

- Indicate how samples were allocated into experimental groups (in the case of clinical studies, please specify allocation to treatment method); if randomization was used, please also state if restricted randomization was applied
- Indicate if masking was used during group allocation, data collection and/or data analysis

Please outline where this information can be found within the submission (e.g., sections or figure legends), or explain why this information doesn’t apply to your submission:

This was not a clinical study. We used a within-subject designed with each participant completed trials in the two experimental conditions, i.e., ‘Training’ and ‘No Training’.

**L502-505:** Each experimental session consisted of 30 seconds of rest, a block of 10 trials in the ‘Training’ condition and a block of 10 trials in the ‘No Training’ condition (Figure 1B). The instruction for each block was presented for 10 s before the block started. The order of training and no training blocks was randomized in each session.

All participants performed the same experimental tasks and within-subject comparisons were performed between two experimental conditions.

**L510-512:** Nine out of 12 participants completed 4 sessions of the task separately with both hemispheres and contralateral arms, and the other three participants only completed the task with the dominant hand for the motor task and the contralateral STN.

For GLMEs, all valid trials from both experimental conditions were included.

**L654-656:** When applying GLME modelling, data from all valid individual trials from all tested hemispheres were considered.

**Additional data files (“source data”)**

- We encourage you to upload relevant additional data files, such as numerical data that are represented as a graph in a figure, or as a summary table
- Where provided, these should be in the most useful format, and they can be uploaded as “Source data” files linked to a main figure or table
- Include model definition files including the full list of parameters used
- Include code used for data analysis (e.g., R, MatLab)
- Avoid stating that data files are “available upon request”

Please indicate the figures or tables for which source data files have been provided:

We have uploaded the data and the code with which one can generate the figures in the main manuscript (**Fig. 2-7 and figure supplements**), as well as the results in **Table II.**
